# Isolation and Purification of a Novel Deca-Antifungal Peptide from Potato (*Solanum tuberosum* L. cv. Jopung) Against *Candida albicans*

**DOI:** 10.3390/ijms13044021

**Published:** 2012-03-23

**Authors:** Jong-Kook Lee, Ramamourthy Gopal, Chang Ho Seo, Hyeonsook Cheong, Yoonkyung Park

**Affiliations:** 1 Research Center for Proteineous Materials, Chosun University, Gwangju 501-759, Korea; E-Mails: seal9669@hanmail.net (J.-K.L.); ramagopa@gmail.com (R.G.); 2 Department of Bioinformatics, Kongju National University, Kongju, South Korea; E-Mail: chseo@kongju.ac.kr; 3 Department of Biotechnology, Chosun University, Gwangju 501-759, Korea; E-Mail: hscheong@chosun.ac.kr

**Keywords:** AFP-J, partial acid digestion, Potide-J, antibiotic agent

## Abstract

In a previous study, an antifungal protein, AFP-J, was purified from tubers of the potato (*Solanum tuberosum* cv. L Jopung) and by gel filtration and HPLC. In this study, the functional peptide was characterized by partial acid digestion using HCl and HPLC. We obtained three peaks from the AFP-J, the first and third peaks were not active in the tested fungal strain. However, the second peak, which was named Potide-J, was active (MIC; 6.25 μg/mL) against *Candida albicans*. The amino acid sequences were analyzed by automated Edman degradation, and the amino acid sequence of Potide-J was determined to be Ala-Val-Cys-Glu-Asn-Asp-Leu-Asn-Cys-Cys. Mass spectrometry showed that its molecular mass was 1083.1 Da. Finally, we confirmed that a disulfide bond was present between Cys^3^ and Cys^9^ or Cys^10^. Using this structure, Potide-J was synthesized via solid-phase methods. In these experiments, only the linear sequence was shown to display strong activity against *Candida albicans*. These results suggest that Potide-J may be an excellent candidate compound for the development of commercially applicable antibiotic agents.

## 1. Introduction

Plants generate a variety of proteins (peptides) that serve to protect against pathogens and invading organisms, include protease inhibitors [1] and antifungal proteins [2–4]. In addition, antifungal peptides and proteins have been purified from a variety of plant species [2–6]. Even though the protection mechanisms of these compounds vary significantly among diverse types of organisms, for example the lack of an adaptive immune response in plants, recent data has suggested that protection strategies of organisms share general features, including the use of a wide variety of small antimicrobial proteins (peptides) as effecter molecules of nonspecific or innate immunity [7,8]. Plants have been shown to make several types of proteins (peptides) that mediate defense against pathogens and invading organisms, including ribosome-inactivating proteins [9], lectins [10], protease inhibitors [11,12], and antifungal proteins [13]. Among these proteins, protease inhibitors are believed to play an important role in the defense against attack by both microorganisms and insects. In addition, these protease inhibitors have been shown to serve as storage proteins and, perhaps, be involved in the regulation of endogenous proteases during seed dormancy [14]. In a previous study, we identified a protease inhibitor, the antifungal protein J (AFP-J), from potato tubers. AFP-J displayed inhibitory activity against human pathogenic fungal strains.

Here, we characterized the antifungal peptide (Potide-J) from AFP-J by HCl digestion. Potide-J was shown to display inhibitory activity against *Candida albicans* and its molecular mass was determined by mass spectrometry. In addition, we analyzed the peptide sequence using automated Edman degradation. Finally, Potide-J was synthesized by solid-phase methods and its antifungal activity was evaluated.

## 2. Results and Discussion

### 2.1. Purification of the Antifungal Peptide, Potide-J

**Figure 1:**
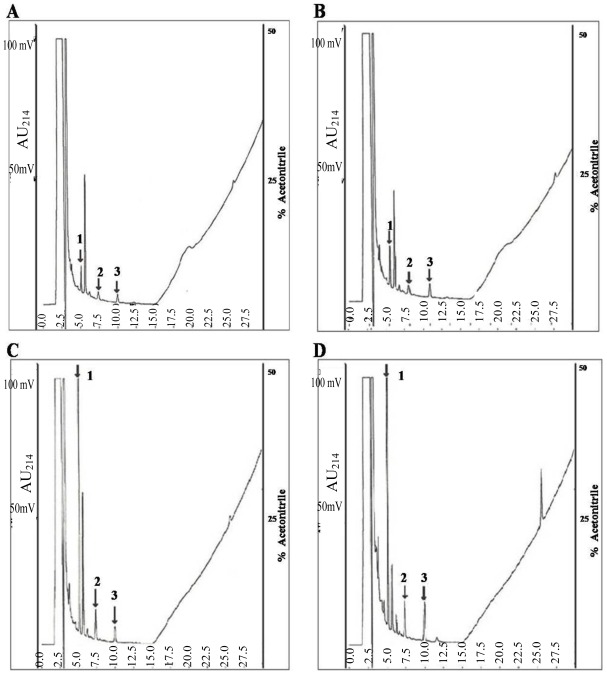
AFP-J protein partial digested with HCl. A sample (10 mg) of AFP-J purified protein was incubated with 1 N HCl at 60 °C for 2–24 h. **A**: 2 h, **B**: 4 h, **C**: 8 h, **D**: 24 h.

The extract of the potato tuber was fractionated on Sephacryl S-100 into unadsorbed fractions (Fraction I and Fraction II) without antifungal activity and an adsorbed fraction (Fraction III) with activity. Fraction III, which displayed antifungal activity, was separated by FPLC using a Superdex 200 prep grade column. The large absorbed peak was then purified by two steps on a C18-reverse phase-high performance liquid chromatography (HPLC). The large adsorbed peak contained a single protein with an approximate molecular mass of 15 kDa [15]. The purified single protein was subjected to HCl digestion, which resulted in three peaks (Figure 1). The protein yields at the various chromatographic steps are shown in Table 1.

### 2.2. Antifungal and Non-hemolytic Effects of Potide-J

**Figure 2:**
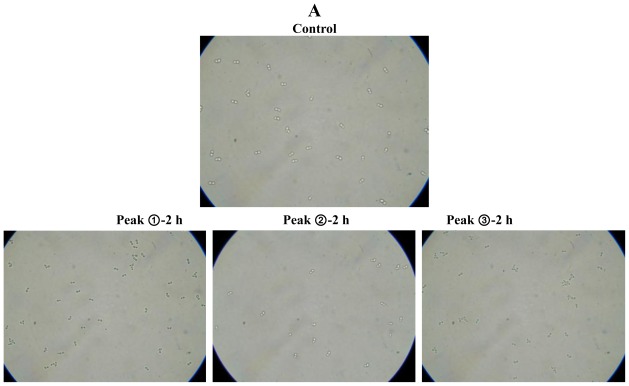

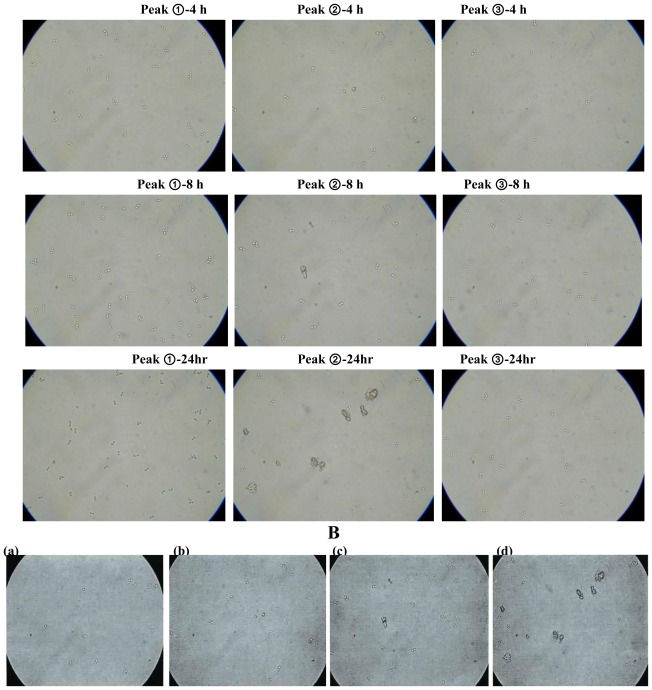
Antifungal activity of the purified peptides digested with 1 N HCl against *C. albicans* (**A**). Antifungal activity of purified peptides against *C. albicans*. Yeast cells were suspended at density of approximately 2 × 10^3^/mL in YPD. Dilutions of the peptide were added, and reaction mixture was incubated for 2, 4, 8 and 24 h at 30 °C. Top row: *C. albicans* cells not treated with the purified peptides. Antifungal activity of the second peak (Potide-J) aganist *C. albicans*. (**B**) **a**: ➁-2 h, **b**: ➁-4 h, **c**: ➁-8 h, **d**: ➁-24 h.

We then examined the antifungal activity of Potide-J against the human pathogenic fungi using the MTT assay (Figure 2). Potide-J was shown to display potent antifungal activity against the human pathogenic fungi *C. albicans*. Light microscopy confirmed that Potide-J strongly inhibited the growth and aggregation of *C. albicans* (Figure 2). The first and third peaks did not display antifungal activity; however, the second peak, which was named Potide-J, prevented the aggregation of fungal cells after 4 h (Peak ➁-8 h, Figure 2A and B-(b)) and after 24 h the fungal cells were highly inhibited (Figure 2A,B-(d)).

The cytotoxicity of Potide-J against mammalian cells was assessed by measuring the lysis of human erythrocytes. In these experiments, Potide-J showed no hemolytic activity (data not shown). These results showed that Potide-J displayed remarkable antifungal activity against human pathogenic fungi but no hemolytic activity.

### 2.3. Protein Identification

**Figure 3:**
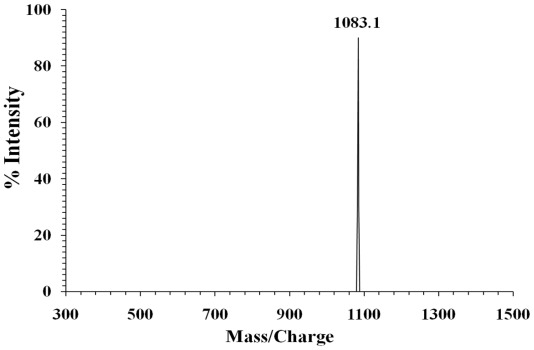
Mass spectrum of Potide-J.

Potide-J had a complete *N*-terminal amino acid sequence of NH_2_-Ala-Val-Cys-Glu-Asn-Asp-Leu- Asn-Cys-Cys. The relative molecular weight of Potide-J was 1083.1 Da, which was directly determine d by MALDI-MS (Figure 3).

**Figure 4:**
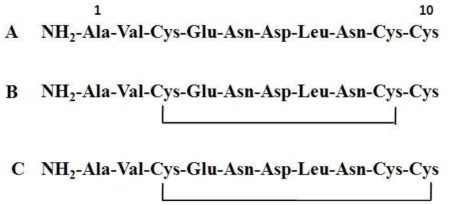
Three synthetic peptides were synthesized. (**A**) linear type sequence, (**B**) peptide with a disulfide bond between Cys3 and Cys9 and (**C**) peptide with a disulfide bond between Cys3 and Cys10.

**Figure 5:**
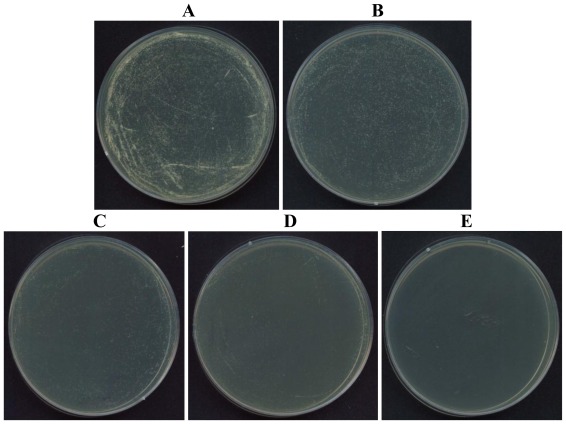
Antifungal activity of synthetic potide-J peptide aganist *C. albicans*. **A**: not treated with peptide, **B**: 7.5 μg/mL, **C**: 15 μg/mL, **D**: 30 μg/mL, **E**: 60 μg/mL.

In addition, we confirmed that whether a disulfide bond was present or not between Cys and Cys in potide-J. Based on this complete *N*-terminal amino acid sequence (NH_2_-Ala-Val-Cys-Glu-Asn-Asp-Leu-Asn-Cys-Cys), three synthetic peptides were synthesized: peptide containing the linear type sequence, peptide with a disulfide bond between Cys3 and Cys9, and peptide with a disulfide bond between Cys3 and Cys10 (Figure 4). The antifungal activity of these synthetic peptides was then evaluated against *C. albicans*. In these experiments, only the linear type (Potide-J) sequence displayed antifungal activity. As shown in Figure 5, Potide-J inhibited the growth of *C. albicans*.

Novel plant antibiotic peptides, including the snakin/GASA (gibberellic acid-stimulated Arabiopsis) family of 12-cystein peptides have been isolated from potato [16]. Novel antibiotic peptides also include shepherdins, which are linear glycine/histidine-rich peptides isolated from the roots of shepherdins purse (*Capsella bursa-pastoris*) [17]. In addition, macrocyclic cystein-knot peptides were also recovered from different plants belonging from the Rubiaceae (coffee and other tropical plants) violacea families when screening for anti-HIV compounds [18].

Potide-J was shown to display potent antifungal activity against human fungal pathogens. In this study, we examined the effect of Potide-J on *C. albicans*, which is the most common cause of oral, esophageal, vaginal, and urinary [19] candidiasis [20], which is found in soil and occasionally is part of the normal flora in human skin. Several antimicrobial peptides have also been purified from potato tubers. For example, a 5-kDa Pseudothionin *Solanum tuberosum* (Pth-St1) was found to be active against bacterial and fungal pathogens of potato such as *Clavibacter michiganensis* subspecies sepedonicus, *Pseudomonas solanacearum* and *Fusarium solani* [21]. Therefore, like these other peptides, Potide-J may have potential therapeutic use of antifungal agent.

Studying plant defense responses and developing new ecofriendly strategies to protect plants against pests and pathogens is currently one of the most dynamic areas of research in plant science. The results obtained in this study suggest that protease inhibitors are involved in the defense response of the host plant against phytopathogens. In addition, we found that these compounds may be useful as effective antimicrobial agents and warrant further study. In addition, they may have the potential to be used as non-cytotoxic clinical agents [15].

## 3. Experimental Section

### 3.1. Potato Tubers

Potato tubers (*Solanum tuberosum* L cv. Jopung) were obtained from the Natural Institute of Highland Agriculture (Kangwon-do, Korea) and were stored at 4 °C in the dark at a relative humidity of 60% for up to 6 months.

### 3.2. Step I: Preparation of AFP-J

Potato tubers were first soaked in distilled water for a few hours and then ground to a fine powder in a coffee grinder. Protein extraction buffer (50 mM Tris-HCl, pH 7.5, 10 mM EDTA, 150 mM NaCl, 1% DMSO, and 0.1% β-mercaptoethanol) was then added. The supernatant was separated by chromatography using a Sephacryl S-100 gel filitration column (2.5 × 95 cm) in 50 mM ammonium bicarbonate buffer (pH 8.0) followed by fast protein liquid chromatography (FPLC) using a Superdex 200 prep grade column with the same buffer. The purity and molecular weight of the fraction with antifungal activity were estimated by sodium dodecyl sulfate-polyacrylamide gel electrophoresis (SDS-PAGE) on a 15% acrylamide gel according to the method of Laemmli and Favre [22].

### 3.3. Step II: Preparation of Potide-J

**Figure 6:**
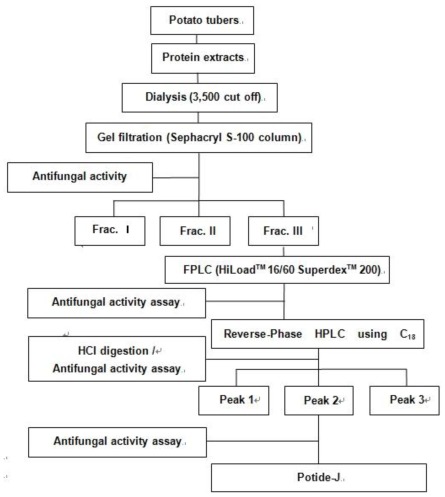
Steps used to purify the antifungal peptide (Potide-J) from potato tubers.

10 mg of purified AFP-J protein was subjected to HCl for 0, 2, 4, 8 and 24 h at 60 °C. After digestion, the loading buffer was immediately added into the samples to terminate digestion and all samples were examined on Reverse-Phase HPLC (RP-HPLC). RP-HPLC was performed in acetonitrile buffer with 0.1% TFA using a linear gradient (40%–80%, 1%/min) [23]. The final peak was separated by RP-HPLC (Figure 6).

### 3.4. Assay for Antifungal Activity

*Candida albica*n*s* (TIMM 1768) was obtained from the Teikyo University Institute of Medical Mycology (TIMM). Microdilution assays to establish minimal inhibition concentration (MIC) values of Potide-J were performed. *Candida albicans* was grown at 28 °C in YPD (2% dextrose, 1% peptone, and 0.5% yeast extract, pH 5.5) for 3 h. Cell densities were counted using a hemocytometer. The fungal cells (2 × 10^3^/well) were seeded in the wells of a flat-bottom 96-well microtiter plate (Greiner, Nurtingen, Germany) containing YPD (100 μL/well). Serial dilutions of the AFP-J solution were added to each well, and the cell suspension was incubated at 28 °C for 24 h. Ten microliters of a 3-(4,5-dimethyl-2- thiazolyl)-2,5-diphenyl-2*H*-tetrazolium bromide (MTT) solution (5 mg/mL) was added to each well, and the plates were incubated at 37 °C for 4 h [24–26]. The absorbance was measured at 570 nm using an Emax microtiter plate reader (Molecular Devices, California, USA). All assays were performed in triplicate. To visualize the fungicidal effect, morphological changes were examined by phase contrast light microscopy using an ECLIPSE TE300 microscope (Nikon, Japan).

### 3.5. Visualize on Agar Plate of Synthetic Peptides

To confirm of Potide-J structure, we designed and synthesized of three types of peptides (linear, disulfide bond between Cys3 and Cys9, and Cys3 and Cys10). To visualize the antifungal activity, the peptides were treated on *C. albica*n*s* and reacted at 28 °C for 18 h. The reacted mixture was spread in YPD agar plate. The agar plate is incubated at 28 °C for overnight.

### 3.6. *N*-terminal Amino Acid Sequencing

For protein sequencing, AFP-J was separated by SDS-PAGE on a 15% acrylamide gel in the presence of 2-mercaptoethanol and then transferred onto a polyvinylidene difluoride membrane (Bio-Rad, Hercules, CA). The protein band was identified by staining with Coomassie brilliant blue, destained with 10% acetic acid, and then excised. The *N*-terminal amino acid sequence was determined from the excised band at the Sequence Centre of the Korea Basic Science Institute (Seoul, Korea).

### 3.7. Mass Spectrometry

MALDI-MS (Matrix-assisted laser desorption ionization mass spectrometry) analysis was performed in the linear mode using a Voyager DE RP instrument (Perseptive Biosystems, Framingham, MA) as described Pouvreau *et al.* [27].

### 3.8. Peptide Synthesis and Purification

All peptides were synthesized using the solid-phase methods with Fmoc (*N*-(9-fluorenyl) methoxycarbonyl)-protected amino acids. 4-Methyl benzhydrylamine resin (Novabio-chem) (0.55 mmol/g) was employed to create the amidated *C*-terminus. At each coupling step, Fmoc-protected amino acid and coupling reagents were added in a 10-fold molar excess relative to the resin concentration. Coupling (60–90 min) was conducted using DCC (dicyclohexylcarbodiimide) and HOBT (1-hydroxy benzotriazole) in NMP (*N*-methyl-2-pyrrolidone). Cleavage from the resin and deprotection of the synthesized peptide were accomplished using a solution of 90% trifluoroacetic acid, 3% water, 1% triisopropylsilane and 2% of 1,2-ethanedithiol, thioanisole, and phenol. After repeated ether precipitation, the crude peptide was purified by reversed-phase preparative HPLC on a Waters 15-μm Deltapak C18 column (19 × 300 mm) using a 0–60% acetonitrile gradient in 0.1% trifluoroacetic acid. The purity of the purified peptide was determined via analytical reversed-phase HPLC using a Vydac C18 column (4.6 × 250 mm, 300 Å, 5 nm). The molecular mass of the peptides was verified using a matrix-assisted laser desorption ionization mass spectrometer (MALDI II, Kratos Analytical Ins.).

## 4. Conclusions

In summary, we isolated Potide-J from the potato tuber (*Solanum tuberosum* L. cv. Jopung) and demonstrated that Potide-J inhibited the growth of human pathogenic fungi cells but displayed no hemolytic activity. Because of this potent antifungal activity and lack of activity against eukaryotic cells, Potide-J holds great promise for use as a candidate compound for the development of novel therapeutic antibiotics.

## Figures and Tables

**Table 1:** Steps in the purification of Potide-J from potato tubers.

Fraction	Yield (mg)
Potato tuber	200,000
Protein Extraction Buffer	85.42
Gel filtration	15.3
FPLC	7.86
First C_18_-HPLC	1.56
Second C_18_-HPLC	0.05
